# Peripartum factors associated with variation in voluntary postpartum hay intake in dairy cows

**DOI:** 10.3168/jdsc.2023-0394

**Published:** 2023-10-06

**Authors:** L.E. Engelking, M. Oba

**Affiliations:** Department of Agricultural, Food and Nutritional Science, University of Alberta, Edmonton, Alberta, Canada T6G 2P5

## Abstract

The objective of this research was to assess variation in postpartum hay intake when offered alongside total mixed ration (TMR) as free choice, and identify factors related to the hay intake. Twenty multiparous cows were fed a closeup TMR (21.5% starch, 39.1% neutral detergent fiber [NDF] on a dry matter [DM] basis). After calving, cows were offered free choice timothy hay (61.6% NDF, 9.6% crude protein) in addition to a fresh cow TMR (26.8% starch, 33.0% NDF) for the first 5 d postpartum. Cows were fed individually with separate mangers for TMR and hay, each offered ad libitum. Prepartum DM intake (DMI) was recorded, and baseline blood samples were collected after calving, but before the first postpartum feeding. Free choice hay intake ranged from 0 to 4.7 kg/d (DM basis) or 0 to 55.2% (% of total DMI). Cows that consumed more hay (% of total DMI) from d 1 through 5 postpartum had lower DMI 2 d before calving (r = −0.63), and greater baseline concentrations of plasma β-hydroxybutyrate (r = 0.60) and serum haptoglobin (r = 0.68). Additionally, hay intake (% of total DMI) from d 1 through 5 postpartum tended to be positively related to baseline plasma fatty acid concentration (r = 0.41). These findings suggest that cows with lower intake before calving and cows with greater ketone production and inflammation at calving may consume more hay, when offered separate from TMR.

## Highlights

• Voluntary hay intake was very variable among fresh cows.
• Cows with lower precalving intake consumed more hay.
• Cows with higher plasma β-hydroxybutyrate concentration at calving consumed more hay.
• Cows with higher serum haptoglobin concentration at calving consumed more hay.

## Graphical Abstract

**Figure:**
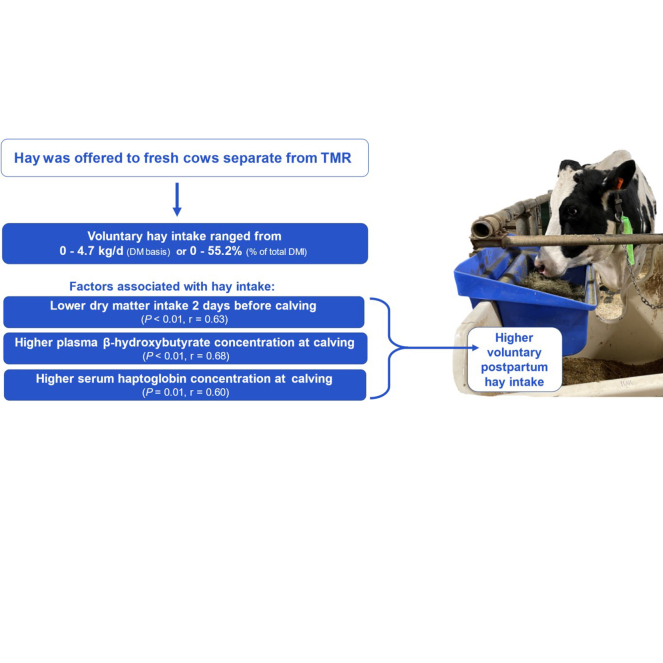
**Summary:** Variation in hay intake among fresh cows and factors related to the hay intake were evaluated. Cows were offered ad libitum hay, separate from total mixed ration (TMR), for the first 5 days in milk. Hay intake was very variable among cows, and was negatively correlated with intake 2 days before calving, and positively correlated with postcalving plasma β-hydroxybutyrate and serum haptoglobin concentration. These findings show that lower precalving intake and a greater concentration of ketone and inflammatory markers are associated with greater voluntary hay intake in fresh cows.

It has been suggested that ruminants have “nutritional wisdom” (Provenza, 1995; Forbes and Provenza, 2000; Miller-Cushon and DeVries, 2017) as they voluntarily adjust dietary intake of fermentable carbohydrates (Villalba and Provenza, 1999) and physically effective fiber (Yang and Beauchemin, 2006), perhaps in a manner to support their nutritional and metabolic needs (Ginane et al., 2015). It has been postulated that animals will adjust feeding behavior to maximize energy intake (Breed and Moore, 2016), which is supported by reports of dairy cattle voluntarily increasing consumption of energy-dense grain compared with forage (Leonardi and Armentano, 2003; DeVries et al., 2008; Greter et al., 2008), with cows experiencing greater negative energy balance sorting to a greater extent for energy-dense particles (Moore and DeVries, 2020). However, in some instances when dairy cows are given the choice between forage and energy-dense feeds, cows will voluntarily increase forage consumption (Yang and Beauchemin, 2006; Kmicikewycz and Heinrichs, 2014), despite the lower energy content in forage compared with grain, and it has been proposed that factors beyond nutritional requirements may influence forage intake in ruminants (Forbes and Kyriazakis, 1995; Miller-Cushon and DeVries, 2017).

Forage intake appears to be very variable in cows, even when cows are fed the same diet and are at similar DIM (Leonardi and Armentano, 2003). The large variation in forage intake of cows with relatively similar nutrient requirements may be due to variable physiology and metabolism of animals (Launchbaugh and Howery, 2005; Manteca et al., 2008); cows may individually adjust the feed type consumed according to individual differences in physiology and metabolism (Cooper et al., 1995; Kyriazakis et al., 1999). Previous studies have evaluated factors that may influence voluntary forage selection in beef calves (Atwood et al., 2001), beef cattle (DeVries et al., 2014a,b) dairy calves (Miller-Cushon et al., 2013a,b; Costa et al., 2016), and acidosis-challenged mid-lactation cows (Keunen et al., 2002; DeVries et al., 2008; Kmicikewycz and Heinrichs, 2014). Havekes et al. (2020) reported that cows sorted for long forage particles in the first week after calving, but research with dairy cows immediately after calving is limited. Understanding factors that influence feed selection and intake is important as it would affect metabolism and health, and selective feed consumption may allow us to better manage individual cow health (Launchbaugh and Howery, 2005; Miller-Cushon and DeVries, 2017), particularly during the transition period when the majority of metabolic disorders occur (Drackley, 1999; Van Saun, 2016). As such, the objective of this research was to assess the extent of variation in hay intake among fresh cows offered free choice hay alongside TMR, and identify factors related to the hay intake.

This study was conducted at a tiestall barn (Edmonton, Alberta, Canada) at the University of Alberta Dairy Research and Technology Centre from January through August of 2021. All procedures were approved by the University of Alberta Animal Care and Use Committee for Livestock (AUP00003716). Twenty multiparous Holstein cows were enrolled in the present study. All cows were fed a closeup TMR (21.5% starch, 16.3% CP, 39.1% NDF, and 32.1% forage NDF on a DM basis; NE_L_ = 1.47 Mcal/kg DM) in individual mangers from 21 ± 3 d before expected calving date until calving. For 5 d after calving, cows were offered free choice timothy hay (1.0% starch, 9.6% CP, 61.6% NDF) in addition to a fresh cow TMR (26.8% starch, 18.1% CP, 33.0% NDF, and 23.4% forage NDF on a DM basis; NE_L_ = 1.63 Mcal/kg DM; barley silage was the sole forage source). Free choice timothy hay was 2.54-cm theoretical length of cut with a particle size distribution of 16.8, 11.8, 33.0, and 38.4% for the first (>19.0 mm), second (8.0–19.0 mm), third (1.18–8.0 mm), and fourth (<1.18 mm) sieves of the Penn State Particle Separator (Kononoff et al., 2003), respectively. Fresh cow TMR had a particle size distribution of 9.6, 31.0, 40.3, and 19.1%, on an as-fed basis, for the first (>19.0 mm), second (8.0–19.0 mm), third (1.18–8.0 mm), and fourth (<1.18 mm) sieves of the Penn State Particle Separator (Kononoff et al., 2003), respectively.

Cows were fed individually with separate mangers for TMR and hay (approximately 99 cm and 61 cm wide, respectively), each offered ad libitum in addition to ad libitum water access. Total mixed ration was fed once daily at 0800 h at 105% to 110% of actual intake of the previous day. Hay was fed daily at 0800 h and was replenished throughout the day as needed. The fresh cow TMR was formulated using the Nutritional Dynamic System (NDS; CNCPS version 6.55, RUM&N) to meet or exceed nutrient requirements for a 650-kg cow producing 31 kg/d of milk with 3.8% milk fat and 3.0% milk protein. Cows were milked twice daily at 0500 and 1600 h.

Hay and TMR offered and refused were weighed daily to calculate daily intake. Samples of free choice hay and closeup and fresh cow TMR ingredients were collected weekly to determine their nutrient content and particle size distribution. Wet feed samples were dried at 55°C for 48 h in a forced-air oven. Wet and dried sample weights were used to calculate DM of feeds, and rations were adjusted if feed DM changed more than 2 percentage units. Dried feed samples were ground with a Wiley mill (Thomas Scientific), using a 1-mm screen, and sent to Cumberland Valley Analytical Services (Hagerstown, MD) and analyzed for DM (AOAC International, 2002; method 930.15), OM (AOAC International, 2002; method 942.05), CP (AOAC International, 2000; method 990.03), NDF (Van Soest et al., 1991), and starch (Hall, 2009). Wet closeup and fresh cow TMR ingredients and timothy hay samples were assessed weekly for particle size distribution using the Penn State Particle Separator according to Kononoff et al. (2003).

Baseline blood samples were collected at 0700 h, before the first feeding of fresh cow TMR and hay at 0800 h; for cows calved after daily feed delivery at 0800 h, the baseline blood samples were collected at 0700 h on the following day. Blood was collected from the coccygeal blood vessel into 2 evacuated tubes (Vacutainer, Becton Dickinson and Co.), one containing sodium heparin to harvest plasma, and the other with no additives to harvest serum. The tube with sodium heparin was placed immediately on ice after collection until centrifuging. The serum tube was kept at room temperature for a minimum of 30 min or until the blood was clotted, whichever was later. Blood was centrifuged at 3,000 × *g* (20 min, 4°C) and plasma and serum were stored at −20°C until analysis. Commercial kits were used to determine plasma concentrations of glucose (Autokit glucose; Wako Chemicals USA Inc.), fatty acids (**FA**; NEFA HR2; Wako Chemicals USA Inc.), and BHB (no. H6501; Roche), and serum concentrations of haptoglobin (**Hp**; Phase Haptoglobin Assay, Tridelta Development Ltd.) and serum amyloid A (Multispecies SAA ELISA Kit, Tridelta Development Ltd.) using a plate reader (SpectraMax 190; Molecular Devices LLC).

Data reported in the present study are from 2 d before the actual calving date until d 5; d 1 was defined as the first day cows were provided fresh cow TMR and free choice hay. Daily hay intake was assessed as kilograms per day and as percent of total DMI, and a Tukey-Kramer adjustment test was used to compare daily hay intake. Hay intake (% of total DMI; 5-d mean values) for each cow was used to evaluate its relationship with DMI at 2 d before calving, and concentrations of plasma metabolites and serum inflammation markers right after calving, but before the first feeding. Statistical analysis was conducted using bivariate regression analysis in JMP 16.1.0 (SAS Institute Inc.), and Pearson correlation coefficients were determined using the MULTIVARIATE procedure. Significance was declared at *P* ≤ 0.05 and tendency was declared at 0.05 < *P* ≤ 0.10. A post hoc power analysis for Pearson correlation was performed using the POWER procedure of SAS 9.4 (SAS Institute Inc.); the sample size of 20 allowed for detection of significant relationships between variables with a correlation coefficient of 0.58 (α = 0.05; 80% power).

In the present study, cows had large variation in daily free choice hay intake, ranging from 0 to 4.7 kg/d. Mean (range) hay intake was 1.17 (0.15–2.8), 1.37 (0–4.7), 0.62 (0–2.5), 0.50 (0–2.4), and 0.58 kg (0–2.1 kg) on d 1, 2, 3, 4, and 5 postpartum, respectively (Figure 1). Hay intake on d 2 was higher than d 3, 4, and 5 (*P* < 0.0001). Mean (range) hay intake (% of total DMI) was 8.8% (1.0–21.1%), 8.0% (0–30.6%), 5.9% (0–47.0%), 6.1% (0–55.2%), and 3.7 (0–16.7%) on d 1, 2, 3, 4, and 5 postpartum, respectively. Intake of fresh cow TMR was 14.1 (6.4–22.1), 13.9 (4.9–24.5), 15.4 (2.8–24.6), 14.7 (0.7–23.6), and 16.2 kg (6.2–26.0 kg) d 1, 2, 3, 4, and 5 postpartum, respectively. Due to variable hay intake, starch content (%) of consumed diets (TMR + hay) varied among cows; mean (range) dietary starch content, on a DM basis, was 24.6 (21.4–26.6), 24.9 (18.9–26.8), 25.3 (14.7–26.8), 25.3 (12.6–26.8), and 25.9 (22.5–26.8) on d 1, 2, 3, 4, and 5 postpartum, respectively. Nonetheless, these data should be interpreted with caution as orts were not analyzed for nutrient composition in the present study, and the starch content of consumed diets was calculated assuming no sorting. Additionally, we acknowledge that particle size of the fresh cow TMR was smaller than intended; thus, less inclusion of physically effective fiber in the fresh cow TMR may have affected voluntary free choice hay intake in the fresh period.

**Figure 1:**
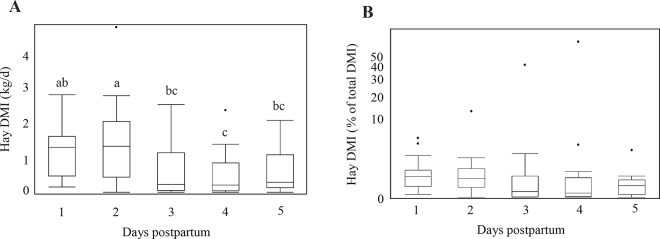
(A) Absolute hay intake (kg/d) and (B) hay intake (% total DMI) for cows offered ad libitum free choice hay for the first 5 d postpartum. Day 1 was defined as the first day cows were provided fresh cow TMR and free choice hay. The horizontal black line within each box denotes the median value; the boxes extend from the first to third quartile, representing the interquartile range; the whisker extending from the bottom of the box represents 1.5 times below the interquartile range; the whisker extending from the top of the box represents 1.5 times above the interquartile range; dots beyond the upper whisker denote outliers.

Free choice hay intake was not associated with baseline concentration of plasma glucose (*P* = 0.64, r = −0.12) or serum amyloid A (*P* = 0.35, r = 0.26). Similarly, postpartum TMR intake was not associated with baseline concentration of plasma glucose (*P* = 0.61, r = 0.12), serum amyloid A (*P* = 0.43, r = −0.19), or Hp (*P* = 0.17, r = −0.32). However, cows with a higher baseline concentration of serum inflammatory marker, Hp (after calving, before fresh cow TMR and hay were fed), consumed more hay (% of total DMI) from d 1 to 5 (*P* = 0.01; r = 0.60; Figure 2), suggesting inflammatory markers at calving are positively associated with voluntary hay consumption in the fresh period. Little research has assessed factors associated with voluntary forage intake in dairy cows, aside from acidosis research; when cows are fed high starch diets and experience acidosis, they are reported to increase consumption of long forage particles (DeVries et al., 2014a), with greater amounts of forage being consumed in cases of more severe acidosis (DeVries et al., 2014b). Although ruminal acidosis is often associated with increased inflammation (Plaizier et al., 2012; Zebeli et al., 2015), in the present study, serum Hp concentration was measured before fresh cow TMR was offered; thus, cows had not yet consumed high starch diets, and thus inflammation may not be related to acidosis and another mechanism may exist. The present study did not measure rumen pH, however, so we cannot definitively say if it influenced hay intake.

**Figure 2:**
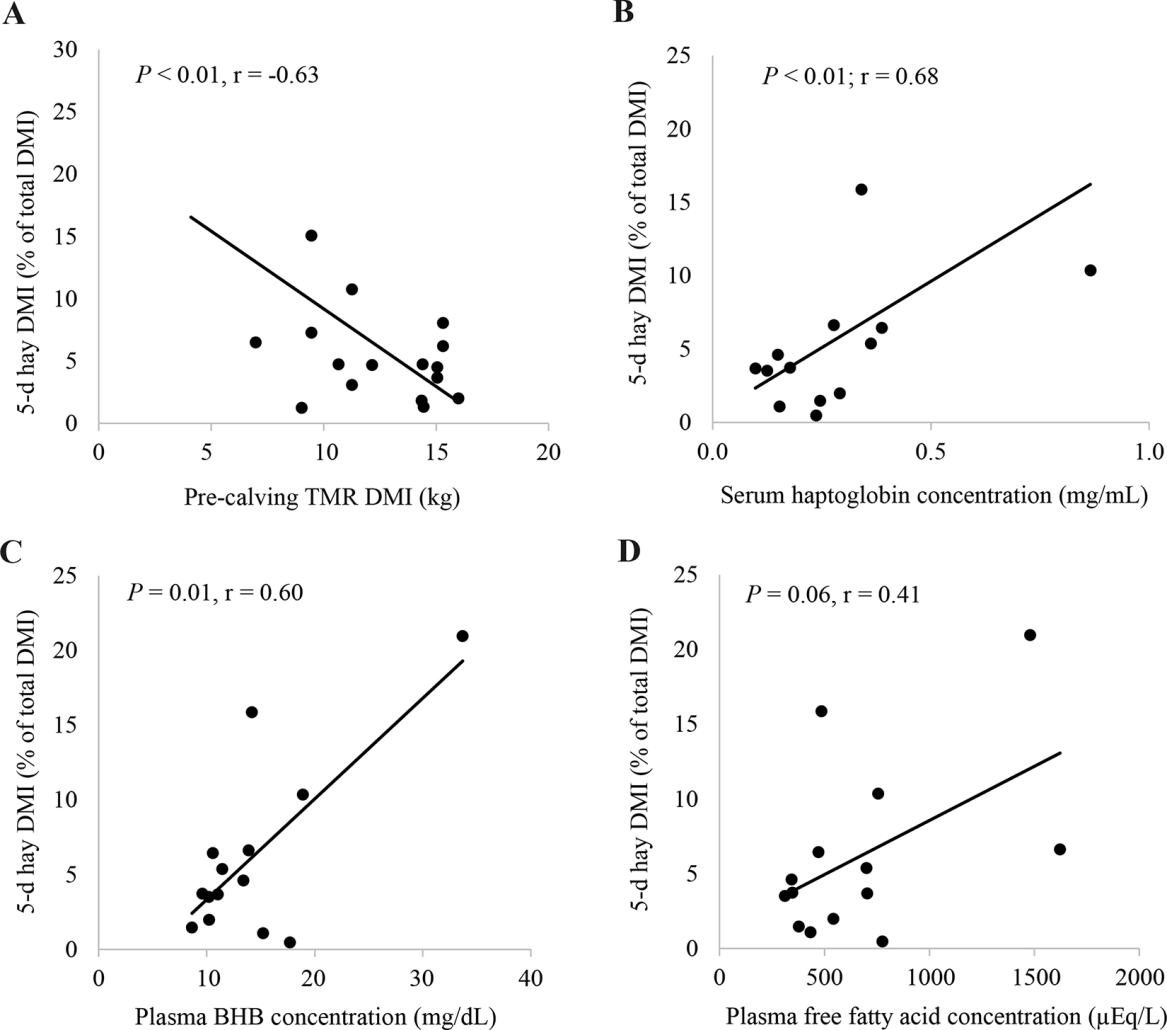
The relationship between 5-d hay DMI (% of total DMI) and precalving DMI (2 d before calving; A), the baseline concentration of serum haptoglobin (B), plasma BHB (C), and plasma fatty acids (D) for cows offered ad libitum free choice hay for the first 5 d postpartum. Baseline plasma and serum samples were collected after calving, but before fresh cow feed and hay were offered to cows.

In monogastrics, it has been suggested that forage intake is motivated by gastrointestinal discomfort (Sueda et al., 2008; Shurkin, 2014), as it may heal gastrointestinal damage and have an anti-inflammatory effect (Fruth et al., 2014). As monogastric animals increase forage intake during gastrointestinal disturbances, it is possible that factors associated with gastrointestinal discomfort, beyond rumen pH, influence forage intake. A similar phenomenon has been proposed in cattle termed “nutritional wisdom,” in the context of cows altering the type of feed consumed based on their internal metabolic state (Provenza, 1995; Forbes and Provenza, 2000; Miller-Cushon and DeVries, 2017), which includes sensorial, metabolic, and physiological signaling (Ginane et al., 2015). Alterations in feed preferences in cows following internal feedback have been described as adaptive because they may allow cows to meet metabolic needs, maintain homeostasis (Ginane et al., 2015), and relieve metabolic discomfort (Gregorini et al., 2015). Feed preference also varies greatly among cows, perhaps due to differences in individual internal metabolic state (Ginane et al., 2015; Gregorini et al., 2015), which may explain the great variation in free choice hay intake in the present study. Providing free choice hay may have allowed cows to adjust their hay intake individually to respond to internal signaling related to inflammation, metabolism, and energy balance; however, additional research is necessary to assess this speculation.

Cows with lower DMI 2 d before calving consumed less TMR (kg/d; *P* < 0.01; r = 0.71) and consumed more hay (% of total DMI; *P* < 0.01, r = 0.63) from d 1 to 5 after calving. In addition, cows with higher baseline plasma BHB concentration (*P* < 0.01; r = 0.68) consumed less TMR (kg/d; *P* = 0.02, r = −0.51) and more hay (% of total DMI) from d 1 to 5. Cows with higher baseline plasma FA concentration tended to consume more hay (% of total DMI; *P* = 0.06; r = 0.41) over the 5-d period; however, baseline plasma FA concentration was not associated with 5-d postpartum TMR intake (kg/d; *P* = 0.18, r = −0.31). Plasma FA and BHB are indicators of energy balance and often increase following reduced intake, leading to fat mobilization and subsequent ketone production (LeBlanc, 2006; Djoković et al., 2017). In the present study, higher BHB and FA were associated with lower precalving DMI; cows with lower DMI 2 d before calving had higher plasma FA (*P* < 0.0001; r = −0.67) and BHB concentration (*P* < 0.001; r = −0.58). In addition, cows with lower DMI 2 d before calving had greater baseline serum Hp concentration (*P* = 0.03; r = −0.64), indicating reduced precalving DMI is associated with increased concentration of inflammatory markers at calving, as described previously by Kuhla (2020). Taken together, greater reductions in precalving DMI may be positively associated with markers of fat mobilization, ketone production, and inflammation, each of which were associated with greater voluntary hay intake in fresh cows in the present study.

It is possible that postpartum hay intake is directly related to precalving intake or postcalving metabolism and inflammation. However, postpartum hay intake might have been influenced by another factor associated with lower precalving DMI and greater postcalving concentration of plasma BHB and FA, and serum Hp. Metabolic disorders are often associated with reduced intake (Sundrum, 2015), increased inflammatory markers (Esposito et al., 2014; Pohl et al., 2015), and increased blood FA and BHB concentration (Ospina et al., 2010). Therefore, reduced precalving intake and increased postcalving inflammation and circulating FA and BHB may occur concurrently with, or as consequence of metabolic disorders, which may increase free choice hay consumption in fresh cows. By providing free choice hay in addition to TMR rather than including hay in TMR, fresh cows experiencing metabolic disorders can alter what they consume based on their needs while other cows consume only TMR to meet high energy demands associated with milk production.

Overall, when free choice hay was provided for 5 d after calving, there was substantial variation in voluntary hay intake among cows. Our findings suggest that reduced precalving intake and greater postcalving ketone production and inflammation are associated with greater voluntary hay intake in fresh cows.
